# EUPATI and Patients in Medicines Research and Development: Guidance for Patient Involvement in Regulatory Processes

**DOI:** 10.3389/fmed.2018.00230

**Published:** 2018-08-17

**Authors:** David Haerry, Cordula Landgraf, Kay Warner, Amy Hunter, Ingrid Klingmann, Matthew May, Wolf See

**Affiliations:** ^1^EUPATI, European Aids Treatment Group, Brussels, Belgium; ^2^EUPATI Swissmedic, Bern, Switzerland; ^3^EUPATI, GlaxoSmithKline, Brentford, United Kingdom; ^4^EUPATI, Genetic Alliance UK, London, United Kingdom; ^5^EUPATI, European Forum for Good Clinical Practice, Brussels, Belgium; ^6^EUPATI, European Patients Forum, Luxembourg, Luxembourg; ^7^EUPATI, BAYER, Berlin, Germany

**Keywords:** patient engagement, patient involvement, guideline, participation, regulatory, medicines development, EUPATI

## Abstract

The importance and merits of greater patient involvement in medicines research and development (R&D) are commonly acknowledged and is thought to offer benefits for all involved parties. It improves discovery, development, and evaluation of new effective medicines, based, among others, on the collaborative identification and understanding of unmet needs, research priorities, optimization of clinical study design, as well as incorporating patient views in regulatory activities. It fosters increased transparency, trust and mutual respect between patients and other stakeholders and applies to all stages of medicines R&D, inclusive of regulation and licensing of medicines and appraisal by health technology assessment (HTA) bodies. In order to be effective and beneficial for all stakeholders, patient engagement as an integral part of medicines R&D needs clear and mutually agreed rules. Existing codes of practice for patient involvement do not comprehensively cover the full scope of patient engagement in all processes related to R&D. One specific aim of the European Patients' Academy on Therapeutic Innovation (EUPATI) was to close this gap through the development of guidance documents for pharmaceutical industry-led medicines R&D, ethics committees, regulatory authorities and health technology assessment (HTA). The guidance in this article covers patient involvement in the regulatory field and draws on the mature “Framework for interaction between the European Medicines Agency and patients and consumers and their organizations.” It expands on the EMA framework, specifically including National Competent Authorities (NCAs). It sets out objectives for patient involvement in medicines regulation and recommends concrete suggested working practices. It is primarily aimed at regulatory authorities wishing to interact with patients or their organizations in their activities but should also be considered by patients/patient organizations planning to collaborate with regulatory authorities.

## Introduction

EUPATI started as a project of the Innovative Medicines Initiative (IMI) Joint Undertaking (2012–2017) and from 1 February 2017 continues as a pan-European public-private partnership program of the European Patients' Forum (EPF). Patient organizations, academia, not-for-profit organizations, and pharmaceutical companies are represented in the partnership. EUPATI focuses on education and training in the general process of medicines development to increase the capacity and capability of patients to contribute to medicines research and development (R&D) with different stakeholders^1^.

The importance of greater patient involvement in medicines research and development (R&D) is commonly acknowledged and is thought to offer benefits for all involved parties (1–4). It helps improving discovery, development, and evaluation of new effective medicines, based, among others, on the collaborative identification and understanding of unmet needs, research priorities, optimization of clinical study design, as well as incorporating patient views in regulatory processes. Patients are in a unique position to describe the outcomes that matter to them, to challenge presumptions about their health aspirations and to inform regulatory processes about the potential positive or negative effects of new and existing health technologies. Beyond this they can give substantial input to the development and provision of adequate and clear information by content, format and language about medicines for patients, as well as the information material for participants in clinical trials. Their contribution is particularly valuable since patients are those directly affected by decisions taken by the regulatory authorities.

Regulatory authorities' activities encompass the evaluation and authorisation of new medicines, and monitoring their safety throughout their lifetime. The European medicines regulatory system is based on a network of around 50 regulatory authorities, the European Commission and EMA and is supported by a large pool of experts drawn from across Europe. EMA and the national competent authorities (NCAs) cooperate and share expertise in the assessment of new medicines and of new safety information. [adapted from European Medicines Agency (5)].

Increasingly, patients are involved in the work of the EMA, including the evaluation of medicines. Such involvement however is not nationwide across the European NCAs but varies considerably between countries and regions, nor is patient involvement seen more broadly in international regulatory processes. Current initiatives by regulatory authorities to engage with patients are mostly in the planning or pilot stage (6, 7).

The involvement of patients with the EMA is determined by European legislation. EMA, its Management Board and its various scientific committees are responsible for developing the relationship between the EMA and its stakeholders (8). These stakeholder relations have evolved over time and the type and degree of interaction varies depending upon the stakeholder group concerned and the type of EMA activity. The EMA Management Board and certain scientific committees include patients and consumers as members. Similar legislative provisions may be lacking at the national level. In their absence, NCAs are developing their frameworks based on EMA experience or independently.

To assess the involvement of patient organizations (9, 10) and patients (11, 12) in regulatory activities, EMA utilized surveys. The results (2008, 2011) and further experience to date indicate that the involvement of patients has resulted in increased transparency, trust and mutual respect between them and other stakeholders (13). It is acknowledged that the patients' contribution to the discovery, development and evaluation of medicines enriches the quality of the evidence and supports quality decision making (14).

In order to be effective and beneficial for all stakeholders, patient engagement as an integral part of medicines R&D needs clear, mutually agreed, rules. Existing codes of practice for patient involvement with various stakeholders do not comprehensively cover the full spectrum of processes related to R&D. One specific aim of the European Patients' Academy on Therapeutic Innovation (EUPATI) was to close this gap through the development of guidance documents for pharmaceutical industry-led medicines R&D, ethics committees, regulatory authorities and health technology assessment (HTA).

The EUPATI guidance in this article covers patient involvement in the regulatory field. It is primarily aimed at regulatory authorities wishing to interact with patients or their organizations in their activities but should also be considered by patients/patient organizations planning to collaborate with regulatory authorities.

The guidance draws on the outcomes of published research, discussions held within the EUPATI project team; two patient involvement workshops with various stakeholders, the results from comprehensive internal and external consultation and in particular on the mature “Revised framework for interaction between the European Medicines Agency and patients and consumers and their organizations” (15). The guidance expands on the EMA framework, specifically including National Competent Authorities (NCAs). An introductory part briefly outlines the background of EUPATI and delineates overarching principles for patient involvement throughout medicines R&D. It includes a set of common values identified by the HTAi in its international consensus-building exercise and adapted to this guidance document. The guidance provides a detailed definition of the term “patient,” sets out objectives for patient involvement in medicines regulation and recommends concrete “suggested working practices.” The guidance is put into the context of current, ongoing debate about patient engagement (PE), with particular regard to their involvement in regulatory authorities' activities. Further steps on how to advance PE, extending the recommendations given in the actual guidance text, are proposed.

The EUPATI guidance document (16) is fully referenced in its online form, but references have been removed from the guidance text here to avoid confusion with citations specific to this article.

## The guidance text

### Guidance for patient involvement in regulatory processes

#### Overarching principles for patient involvement throughout the medicines research and development process

The European Patients' Academy (EUPATI) is a pan-European Innovative Medicines Initiative (IMI) project of 33 organizations with partners from patient organizations, universities, not-for-profit organizations, and pharmaceutical companies. Throughout EUPATI the term “patient” references all age groups across conditions. EUPATI does not focus on disease-specific issues or therapies, but on process of medicines development in general. Indication-specific information, age-specific or specific medicine interventions are beyond the scope of EUPATI and are the remit of health professionals as well as patient organizations. To find out more visit www.eupati.eu/.

The great majority of experts involved in the development and evaluation of medicines are scientists working both in the private and public sector. There is an increasing need to draw on patient knowledge and experience in order to understand what it is like to live with a specific condition, how care is administered and the day-to-day use of medicines. This input helps to improve discovery, development, and evaluation of new effective medicines.

Structured interaction between patients of all age groups and across conditions, their representatives and other stakeholders is necessary and allows the exchange of information and constructive dialog at national and European level where the views from users of medicines can and should be considered. It is important to take into account that healthcare systems as well as practices and legislation might differ.

We recommend close cooperation and partnership between the various stakeholders including healthcare professionals' organizations, contract research organizations, patients' and consumers' organizations^*^^2^, academia, scientific and academic societies, regulatory authorities and health technology assessment (HTA) bodies and the pharmaceutical industry. Experience to date demonstrates that the involvement of patients has resulted in increased transparency, trust and mutual respect between them and other stakeholders.

It is acknowledged that the patients' contribution to the discovery, development and evaluation of medicines enriches the quality of the evidence and opinion available (1).

Existing codes of practice for patient involvement with various stakeholders do not comprehensively cover the full scope of research and development (R&D). The EUPATI guidance documents aim to support the integration of patient involvement across the entire process of medicines research and development.

These guidance documents are not intended to be prescriptive and will not give detailed step-by-step advice.

EUPATI has developed these guidance documents for all stakeholders aiming to interact with patients on medicines research and development (R&D). Users may deviate from this guidance according to specific circumstances, national legislation or the unique needs of each interaction. This guidance should be adapted for individual requirements using best professional judgment.

There are four separate guidance documents covering patient involvement in:

- Pharmaceutical industry-led medicines R&D
- Ethics committees
- Regulatory authorities
- Health technology assessment (HTA).

Each guidance suggests areas where at present there are opportunities for patient involvement. This guidance should be periodically reviewed and revised to reflect evolution.

This guidance covers patient involvement in the regulatory field and draws on the mature “Revised framework for interaction between the European Medicines Agency and patients and consumers and their organizations.”

The following values are recognized in the guidance, and worked toward through the adoption of the suggested working practices. The values are:

**Table:** 

Relevance	Patients have knowledge, perspectives and experiences that are unique and contribute significantly to essential aspects of regulatory activities.
Fairness	Patients have the same rights to contribute to the regulatory activities as other stakeholders and have access to knowledge and experiences that enable effective engagement.
Equity	Patient involvement in regulatory activities contributes to equity by seeking to understand the diverse needs of patients with particular health issues, balanced against the strict requirements of regulatory legislation and guidelines.
Legitimacy	Patient involvement facilitates those affected by regulatory decisions to participate in regulatory activities; contributing to the transparency, accountability and credibility of the decision-making process.
Capacity building	Patient involvement processes address barriers to involving patients in regulatory activities and build capacity for patients and regulatory authorities to work together.

All subsequently developed guidance should be aligned with existing national legislation covering interactions as stated in the four EUPATI guidance documents.

#### Disclaimer

**EUPATI has developed** this guidance for all stakeholders aiming to interact with patients on medicines research and development (R&D) throughout the medicines R&D lifecycle.

These guidance documents are not intended to be prescriptive and will not give detailed step-by-step advice. This guidance should be used according to specific circumstances, national legislation or the unique needs of each interaction. This guidance should be adapted for individual requirements using best professional judgment.

Where this guidance offers advice on legal issues, it is not offered as a definitive legal interpretation and is not a substitute for formal legal advice. If formal advice is required, involved stakeholders should consult their respective legal department if available, or seek legal advice from competent sources.

EUPATI will in no event be responsible for any outcomes of any nature resulting from the use of this guidance.

The EUPATI project received support from the Innovative Medicines Initiative Joint Undertaking under grant agreement n° 115334, resources of which are composed of financial contribution from the European Union's Seventh Framework Programme (FP7/2007-2013) and EFPIA companies.

#### Scope

This European guidance covers the interaction between patients and medicines regulatory authorities in relation to medicines for human use. “Patients” can be individual patients or their careers, or representatives from patient organizations with relevant expertise. Regulatory authorities include both National Competent Authorities (national regulatory authorities) and the European Medicines Agency (EMA). Patients' organizations are not-for-profit organizations that have an interest in patient care, and where patients represent a majority of members in governing bodies.

**Figure 1:**
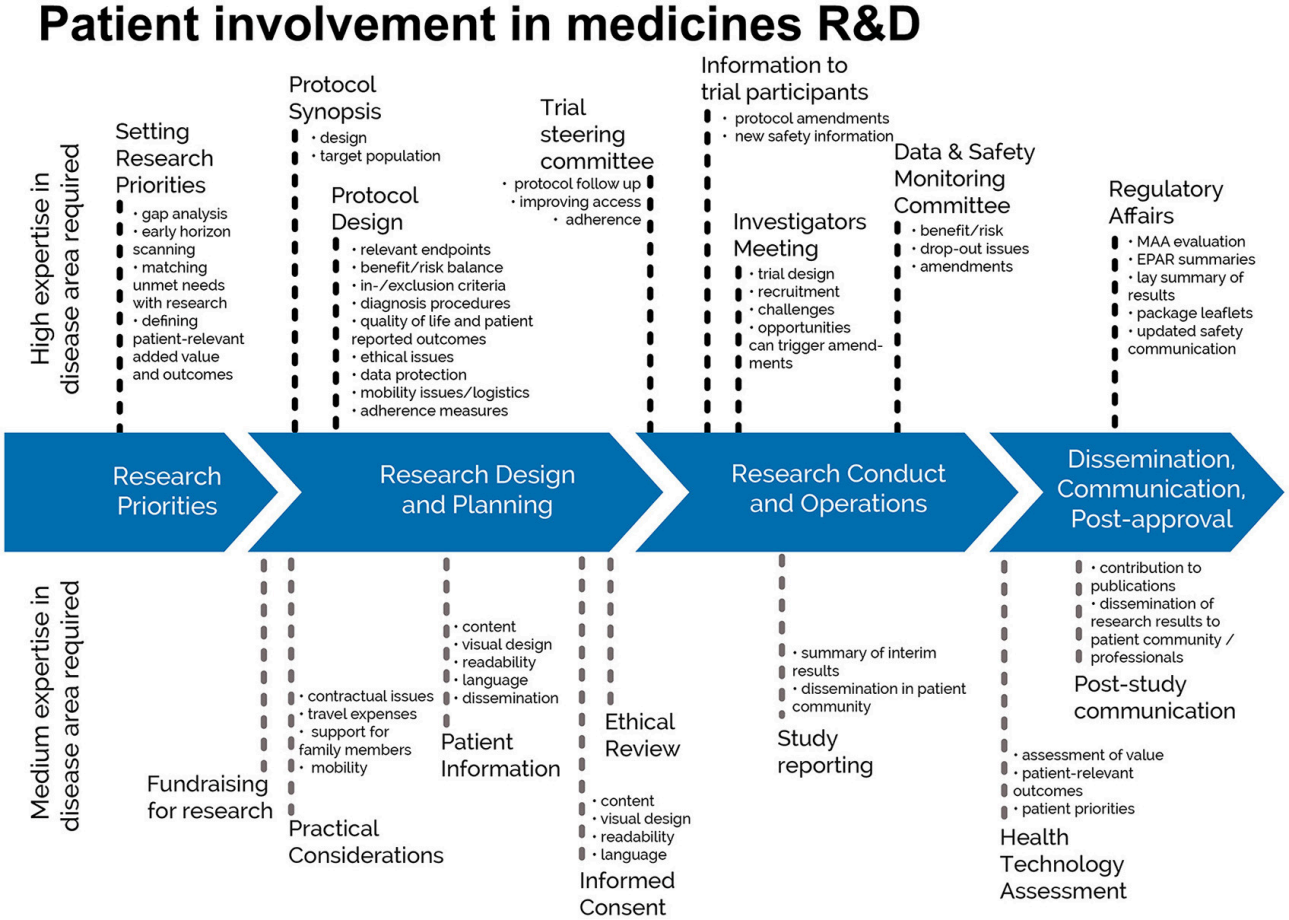
Patient involvement in medicines R&D. Patients can be involved across the process of medicines R&D. This diagram created by Geissler, Ryll, Leto, and Uhlenhopp identifies some existing areas in which patients are involved in the process. It distinguishes between the level of expertise in a disease area that is required and the different areas where involvement can take place.

The guidance focuses on involvement, and excludes the scientific collection of patient perspectives (i.e., quantitative and qualitative systematic research on the psychosocial impact of diseases and treatments). Figure 1 indicates where patients can be involved currently throughout the medicines R&D lifecycle; however this is not meant to limit involvement, and opportunities may change and increase over time.

#### Defining “patient”

The term “patient” is often used as a general, imprecise term that does not reflect the different types of input and experience required from patients, patient advocates and patient organizations in different collaborative processes.

In order to clarify terminology for potential roles of patient interaction presented in this and the other EUPATI guidance documents, we use the term “patient” which covers the following definitions:

- “Individual Patients” are persons with personal experience of living with a disease. They may or may not have technical knowledge in R&D or regulatory processes, but their main role is to contribute with their subjective disease and treatment experience.
- “Carers” are persons supporting individual patients such as family members as well as paid or volunteer helpers.
- “Patient Advocates” are persons who have the insight and experience in supporting a larger population of patients living with a specific disease. They may or may not be affiliated with an organization.
- “Patient Organization Representatives” are persons who are mandated to represent and express the collective views of a patient organization on a specific issue or disease area.
- “Patient Experts,” in addition to disease-specific expertise, have the technical knowledge in R&D and/or regulatory affairs through training or experience, for example EUPATI Fellows who have been trained by EUPATI on the full spectrum of medicines R&D.

There may be reservations about involving individual patients in collaborative activities with stakeholders on grounds that their input will be subjective and open to criticism. However, EUPATI, in line with regulatory authorities, instills the value of equity by not excluding the involvement of individuals. It should be left to the discretion of the organization/s initiating the interaction to choose the most adequate patient representation in terms of which type of patient for which activity. Where an individual patient will be engaged it is suggested that the relevant patient organization, where one exists, be informed and/or consulted to provide support and/or advice.

The type of input and mandate of the involved person should be agreed in any collaborative process prior to engagement.

#### Rationale for the guidance

The extent of patient involvement in regulatory issues varies considerably between countries and regions in Europe.

The EMA has interacted with its stakeholders since its creation in 1995. These stakeholder relations have evolved over time and the type and degree of interaction varies depending upon the stakeholder group concerned and the type of EMA activity. The EMA Management Board and certain scientific committees include patients and consumers as members.

The benefit of stakeholder involvement experienced by the EMA has resulted in several national regulatory bodies implementing a framework for involvement of patients at national level too. Most national regulators draw on the EMA experiences. The involvement of patients with the EMA is determined by European legislation (2). EMA, its Management Board and its various scientific committees are responsible for developing the relationship between the EMA and its stakeholders. The European legislation defines:

- Direct interaction between the EMA and patients' and consumers' organizations, through the Patients' and Consumers' Working Party (PCWP),
- The framework for providing clear and useful information to these organizations.
- Specific forms of interaction, e.g., patients' membership in the EMA Management Board, the Committee for Orphan Medicinal Products (COMP), the Pediatric Committee (PDCO), the Committee for Advanced Therapies (CAT), Scientific Advice/Protocol Assistance procedures with the Scientific Advice Working Party (SAWP) and the Pharmacovigilance and Risk Assessment Committee (PRAC).
- In addition, the EMA has put in place methods to collect patients' input through direct consultation.

The experience acquired to date demonstrates that the participation of patients in EMA activities has resulted in increased transparency and trust in regulatory processes and mutual respect between regulators and the community of patients and consumers. The experience confirms the importance for EMA to continue supporting and facilitating patient contribution to its work.

Similar legislative provisions may be lacking at the national level. In the absence of legal provisions, National Competent Authorities are developing their frameworks on EMA experience or are developing a framework on their own. Key elements to consider for such a framework include:

- Define the role of patients in the interaction
- Include proposals on involving patients in specific institutional processes
- Develop a training programme
- Consider a concept for expert compensation, applying to all stakeholders
- Continuously evaluate the interaction for further improvements and collaborate among agencies with patients to establish and standardize methods and practices.

Any framework needs to be reviewed on a regular basis.

#### Objectives of patient involvement in medicines regulation

Streamlining the interactions with patients, and focusing on areas where mutual benefit can be anticipated, are two underlining principles to consider when implementing a framework.

The aim should be further building of transparency and trust with patient communities through their active engagement **(participation-consultation-information)**. In order to achieve this goal, specific objectives should be met, such as:

- Supporting the regulator to access real-life experiences of diseases and their management and to obtain information on the current use of medicines. This will contribute to understanding the value, as perceived by patients, of the scientific evidence provided during the evaluation process for the purposes of benefit/risk decision-making.
- Ensure that patients and their representative organizations are listened to, consulted and involved in the development of policies and plans;
- Enhance patient organizations' understanding of the mandate and role of the regulator within the context of the development, evaluation, authorisation, monitoring and provision of information on medicines;
- Optimize communication tools (on content and delivery) to facilitate and encourage the cascade of information to the constituencies of patient organizations (i.e., to reach out to individual patients) with the aim of supporting their role in the safe and rational use of medicines;
- Facilitate participation of patients in benefit/risk evaluation and related activities, to capture patients' values and preferences and obtain information on the current use of medicines and their therapeutic environment, all along the lifecycle of medicines development, from early development throughout evaluation and post-marketing surveillance.

Achieving these objectives will necessitate close collaboration between the regulatory authorities, national ministries of health, and other relevant stakeholders, as well as an active participation and good interaction with patients, healthcare professionals and their representative organizations.

#### Suggested working practices (adapted from the EMA framework of interaction)

Based on experience of the EMA at European level, patients can participate in the regulatory authority's activities as:

- **Members (and alternates)** of some of the regulatory authority's (scientific) committees or working groups and, in case of the EMA, of the EMA's Management Board (formally appointed by the EU Institutions).
- Individual **experts**.
- **Representatives of a specific organization**, to be consulted and participate in discussions to express the views of the organization on a specific issue.
- Occasionally **observers** in certain aspects of the EMA's or regulatory authority's work.

Regulatory authorities should establish eligibility criteria.

When patients participate in regulators' activities as individuals and not as representatives of their organization, they should declare any interests and abide by the regulator's code of conduct as any other experts. In addition, the organizations involved with the regulator should be fully transparent with regard to their activities and funding sources.

In order to achieve the objectives identified above, the following six elements should be considered as critical:

- **A network of patient organizations (potentially in collaboration with other regulatory authorities)** The **network of patient organizations** allows the regulator to build up consistent and targeted interactions with a broad group of organizations with a diverse range of expertise and interests. Selection criteria should apply. Such criteria should ensure that the regulator establishes contact with the most suitable organizations representing patients in a transparent manner. Within a network, the criteria should be harmonized.
- **A forum of exchange with patient organizations established within the regulatory authority** This is a platform for dialog and exchange with patient organizations on relevant issues concerning medicines for human use and when relevant medical devices; through it the regulator will inform and will obtain feedback and contribution from patients on various regulator's initiatives. It includes a balanced representation of the different types of patients as well as organizations representing special and vulnerable populations not well represented in medicines development such as older people and women. It should provide a forum to further identify gaps and priorities in the overall interaction.
- **A pool of individual patients acting as experts in their disease and its treatment to facilitate patient involvement in medicines evaluation and information** The creation of the pool of experts will enable the regulator to quickly and efficiently identify patients who can be involved in product-related activities, review of product information and communication material.
- **Interaction particularly in the field of communication** This will provide a valuable contribution to support the existing structures for information dissemination to the public. Furthermore, collaboration in this area will promote the provision of validated and up-to-date information to patients on the benefits and risks of medicines and contribute to the preparation and dissemination of clear messages on the safe and rational use of medicines intended to reach the public. Any information material to patients should be reviewed by patient representatives to improve readability and appropriateness of language and content.
- **A program of actions for capacity-building, focusing on training and raising awareness about the regulatory system** For their contribution to be meaningful, patients must have an understanding of the regulator's mandate as well as the patient's expected role in the evaluation process. A training program should be available. Some patient organizations or other collaborative projects have developed their own training material in order to empower patients to play a recognized advocacy role.
- **Financial support** Financial support should be provided to patients contributing to the regulator's activities. This would represent an acknowledgment of the work they do while promoting their independence. Patients should be recognized as experts and treated according to the same standards as any other experts, also with regard to compensation. Sometimes, patients may need additional assistance to ensure they are able to participate.

#### Defining the interaction

Prior to each interaction it is recommended to mutually agree on (where applicable):

- The objective of the activity involving patients and/or areas of common interest to establish an agreed structured interaction, providing all parties with necessary protection with regards to independence, privacy, confidentiality and expectations.
- The type of input and mandate of the involved person
- The tools and methods of interaction, e.g., frequency of meetings, ground rules, conflict resolution, compensation, evaluation.
  ⁰ The method of interaction (meetings, telephone discussions, etc.) should be discussed and mutually agreed, with convenience for patients/patient organizations as the main priority. If the interaction requires in person meetings or the development and delivery of events, these should follow existing codes of conduct, in terms of appropriate venue/location and the level of hospitality provided.
  ⁰ When events are organized, the ability of any intended patient audience to attend should be considered, with appropriate measures taken to enable accessibility, assisted travel and entry into the event.
- Desired patient and patient partner organization to foster long-term working relationships, with independence ensured.
- The profile of the type of patient(s) or patient representative/s to be involved and the number.
- How activity outputs will be used.
- How and when the patient/s involved will be informed of outcomes.
- Contractual terms and conditions including consent and confidentiality as well as agreement on the interaction itself (type of meeting, frequency, compensation).
- Other elements according to the specific activity.

#### Patient identification/interaction

There are many ways to identify patients to be involved in an interaction. The main routes are through:

- Existing patient organizations
- EUPATI or similar project
- Advertising opportunities for patient participation
- Open call
- Existing relationships with healthcare providers, hospitals and researchers and other agencies
- Unsolicited requests previously made by interested parties
- Existing advisory boards/groups (e.g., Patients and Consumers Working Party at the EMA, EFPIA Think Tank)
- Third party agencies

#### Eligibility criteria

##### Patient organizations

To increase transparency of patient involvement, agencies and patient organizations should plan to publicly disclose their collaborative activities on an annual basis. Individual patient names can be disclosed when the person is part of a generic advisory council but in other instances names should not be disclosed.

Patient organizations shall be committed to take an active part in the interaction with a regulatory authority.

The organizations shall be established in a Member State of the European Union (EU) or of the European Economic Area (EEA), and shall fulfill the following criteria:

Criteria for patient organizations for the interaction with regulatory authorities.

**Table:** 

Legitimacy:	The organization shall have statutes registered in one of the Member States of the EU/EEA. If it is an international organization not registered in an EU/EEA Member State, additional information needs to be provided demonstrating EU focus and activities.
Mission/objectives:	The organization or individual patient expert shall have their mission/objectives clearly defined and should agree to have these published on the regulatory authorities website.
Activities:	The organization shall have, as part of its activities, a specific interest in medicinal products (and when relevant medical devices) which should be documented (e.g., through a report published on the organization or individual person's website).
Representation:	The organization shall be representative of patients throughout the EU/EEA or at the relevant national level. Organizations already registered at Community level, e.g., in the EU Health Forum, the Council of Europe, are considered to adequately represent patients or for involvement in medicines regulatory activities. In case of a lack of European associations for a specific disease or treatment area, the involvement of national organizations may be considered, although preference will be given to European wide-associations. International organizations can also be considered for eligibility as long as they have a European focus and representation, including EU/EEA based office(s).
Structure:	The organization should have governing bodies which are elected by their members, who shall be patients, their carers, or their elected representatives.
Accountability and consultation modalities:	Statements and opinions of the organization should reflect the views and opinions of its members and adequate consultation procedures with those members should be in place. In particular, the organization should ensure that the appropriate flow of information is in place to allow dialog both ways: from and toward its members.
Transparency:	The organization shall disclose to the regulatory authority its sources of funding both public and private by providing the name of the bodies and their individual financial contribution, both in absolute terms and in terms of overall percentage of the organization budget. Any relationship with corporate sponsorship should be clear and transparent. This information shall be communicated to the Agency on an annual basis. In the case of umbrella organizations the list of member associations should be made available to the agency. The organization shall publish on the organization website the registered statutes, together with financial information including its source of funding both public and private, and information on their activities. The organization shall follow a code of conduct/policy regulating its relationship with and independence from the sponsors. The regulatory authority, will evaluate the financial information according to a transparent pre-set regulation.

#### Compensation

It should be recognized that in many situations patients involved in activities do so voluntarily either as an individual but also when a member of an organization. Consideration should therefore be given to:

- compensate for their total time invested plus expenses.
  ⁰ any compensation offered should be fair and appropriate for the type of engagement. Ideally travel costs would be paid directly by the organizing partner, rather than being reimbursed.
- cover the costs incurred by patient organizations when identifying or supporting patients for involvement in activities (i.e., peer support groups, training and preparation)
- help organize the logistics of patient participation, including travel and/or accommodation.

Compensation also includes indirect benefits in kind (such as a patient organization providing services free of charge) or any other non-financial benefits in kind provided to the patient/patient organization (such as training sessions, the setting up of web sites).

#### Written agreement

At a minimum a written agreement should clearly define: a description of the activity and its objectives, the nature of the interaction during the activity, consent (if relevant), release, confidentiality, compensation, data privacy, compliance, declaration of conflict of interest, timelines. Interaction may only proceed on the basis of a written agreement that at a minimum spells out the basic elements of the collaboration (e.g., rules of engagement, compliance, intellectual property, financial payments). Care should be taken so that written agreements are clear and do not limit appropriate knowledge sharing.

#### Implementation and monitoring

A patient involvement framework can be introduced step-by-step and/or following a pilot phase where appropriate. After full implementation, when patients are involved both in general and product specific issues and there is an established pool of organizations and patients as individual experts as well as for fora for interaction, a public annual report on interactions should be prepared including:

- an analysis of indicators (to be defined for the type of interaction) assessing the usefulness of the interactions
- feedback received from patients and their representative organizations through targeted surveys
- feedback received from the regulatory authority itself
- an overview of the activities where organizations and patients as individual experts have been involved
- a suggested way forward, including a strategy for future patients' interaction, is recommended to be proposed.

**Appendices to the guidance are available in the online version of the guidance document (****16****)**

The EUPATI guidance document on patient involvement in regulatory processes was first released in 2016. Further context for patients wishing to better understand medicines regulation is provided by online articles and a recorded webinar on the EUPATI website ^3^,^4^,^5^,^6^.

## End of guidance text

## Discussion

The involvement of patients as valued partners in medicines R&D has gained momentum in the past years and their contribution in collaboration with different stakeholders is recognized. For patient engagement in regulatory authorities' activities the EMA states “The added value of having patients and consumers in the scientific committees is to bring a unique and critical input based on their real-life experience of being affected by a disease and its current therapeutic environment. This element fills a gap that other committee members (so-called scientific experts) cannot fill” (17). Nevertheless, more needs to be done to anchor patient involvement firmly in the process, from research and discovery through development to authorisation and HTA, in essence, covering the whole lifecycle of a medicinal product (2, 4, 18, 19). This should occur in a structured way within a “master framework” (4) or a “widely accepted model or a framework” (20) to make it meaningful and effective. The EUPATI guidance documents provide the first instance of such an overarching framework for key stakeholders in medicines R&D.

The EUPATI guidance documents do not offer detailed step-by-step instructions or provide templates, although this was frequently asked for during the consultation period. This is intended to allow users to adapt to concrete requirements of an interaction and/or national legislation and applies specifically to the regulatory guidance document: national legislation, if covering this area at all, may set (very) tight limits on the possibility to involve patients in regulatory activities of an NCA.

In order to structure patient participation, the EMA has developed and continuously refines/strengthens their “framework for interaction between the European Medicines Agency and patients and consumers and their organizations” (last revision 2014) (15). While the involvement of patients with the EMA is determined by European legislation (8) such legislation is incidental or absent at the national level. Notwithstanding this, several NCAs are planning or piloting patient engagement activities and draw on the EMA experience. Their number, however, is relatively small as indicated by an EMA survey to NCAs in 2015, which received responses from 15 NCAs. Of these respondents, 13 reported interactions with patients to varying degrees and with only a few formal provisions (21). Establishing Europe-wide legislation requiring patient involvement in regulatory authorities' activities would most likely facilitate the process, as seen with the EMA. The EUPATI regulatory guidance document includes NCAs in an attempt to at least provide the frame for a common approach for those NCAs considering patient engagement in their activities, even if not legally obliged to do so.

The EUPATI guidance document requires defining the profile of the type of patient(s) or patient representative/s and contains eligibility criteria for patient organizations to be involved in regulatory authorities' activities. For their contribution to be meaningful, patients should have a certain level of knowledge about the regulatory and R&D process, an understanding of the regulatory authority's mandate as well as their expected role in an interaction.

In this context a few points should be critically considered. To include experiential knowledge of patients in the process of decision making can be challenging for both patients and scientific experts. Regulatory experts have a common knowledge base and a common language and patient experience and preferences are not usually incorporated in the scientific discourse, it is “ignored knowledge.” Bringing this to the process is further complicated by unequal power relations. While patients are challenged to legitimize their input and find ways to demonstrate credibility, experts, who recognize the value of patient involvement, may experience problems when they have to translate experiential knowledge to evidence based language and transform individual statements to statements usable for decision making (adapted from 20). Likewise, a patient's personal expectations may pose a challenge to their involvement as an “expert” in the regulatory process. This is especially so when the topic concerns their specific disease, and they are required to put their personal aspirations in second place to the more overarching questions pertaining to the medicinal product under discussion.

In a paper by Borup et al. (4) the idea of a “professional” patient representative with a recognized (possibly certified) level of education was proposed. However, with increased professionalization, implying the patient being taught in a medical/academic/legislative language, it was feared that the patient might turn from patient into a “professional.” This, it was argued, might influence the patients' position and opinion, thereby decreasing their ability to genuinely represent the patient view and challenge conventions.

This point is well illustrated by a discussion of patient and public involvement in a paper by Ives et al. addressing a possible paradox in patient and public involvement (PPI); the “PPI paradox” (22). In brief it says: the value of patient and public involvement is in the “lay” perspective on research. Efforts to access this expertise can, however, jeopardize the “lay status” of the patient or citizen involved. Patients and citizens who are involved at a sufficiently high level need adequate training to be able to contribute substantially to the process. But once a “lay person” undergoes training, and becomes familiar enough with research to be meaningfully involved, their “lay” status is compromised and they become “more expert.” And even though they can still contribute to the process in a way that is informed by their own experience of illness, their “lay” perspective is at risk of being “tamed” to make it more congruous with that of the professional. A critique on this idea came from Staley, who argued that the views of the lay person are complementary to those of the technical experts. Lay people, he maintains, know what research would help them, how to make participation a positive experience and how best to communicate the findings to a lay audience (23).

Knowledge and expertise requirements were discussed intensely when preparing the guidance document. In line with the intent to formulate the guidance with enough room for users to adapt its usage to specific circumstances and demands of an interaction, it was decided to refrain from specifying required graded knowledge levels for different kinds of engagement but rather define patient profiles according to levels of expertise and experience. However, there was general agreement that the role of the patient should be distinct from that of the genuine (i.e., naïve) “lay person.” Being “concerned,” patients bring different backgrounds and views to the table than lay persons. It was equally noted that the better education and training of patients were, the better they would be equipped to participate in regulatory deliberations. This view is confirmed by the experience made by the EMA when involving patient organizations in the readability review for package leaflets and EPAR summaries (personal communication).

The EUPATI guidance document therefore stipulates that a program of actions for capacity-building, focusing on training and raising awareness about the regulatory system should be available. Some patient organizations and other collaborative projects have developed their own training material, in order to educate and empower patients to play a recognized advocacy role. A prime example is the modular blended learning course on medicines R&D and a web-based toolbox on medicines R&D developed by EUPATI.

It is a demanding task to find knowledgeable patients who understand the methodological problems, can provide representative opinions and feel comfortable in debating with scientific experts. The number of patients with this skill set is small and will further diminish as medical progress moves toward ever smaller cohorts of well characterized patients with a specific disease. Moreover, if overly stringent rules of participation were in place because of suspected conflicts of interest due to patients' affiliations, e.g., with industry, this would decrease the number of available knowledgeable patient experts even further. Legal constraints and a certain degree of competition between patient organizations, specifically on a national level, might heighten the problem of identifying suitable patients for collaborative work with regulators.

Possible conflict of interest (COI) is especially important when interacting with regulatory authorities. The guidance document addresses COI insofar, as a clear statement on possible conflicts of interest is required in the “Written agreement” section. The format may vary, but in essence users of this guidance document should observe the criteria formulated by EMA for patient and consumer organizations and their members actively involved in EMA activities (17, 24). The issue of COI is crucial since an appreciable number of patient organizations accept pharmaceutical industry funding to support their activities. Arguments in the literature are controversial; some state that patient organizations are able to defend their independence from the influence of any sponsor, be they public or private (25), others argue against on grounds that the autonomy of patient organizations would be jeopardized (26, 27). It should not remain unmentioned that some patient organizations have experienced problems regarding their involvement with these policies, which have made some patient representatives no longer eligible.

## Conclusion

The extent of patient involvement in regulatory activities, especially with national regulatory authorities, continues to vary widely across Europe. Establishing an overarching legal framework to determine the extent and provisions for patient engagement with NCAs would facilitate patient involvement in regulatory activities and should be pursued.

Strengthening the education and training for patients and patient representatives in medicines R&D and specifically regulatory processes is paramount to increase the number of adequately prepared patients/patient representatives for involvement in the work of regulatory authorities.

The EUPATI regulatory guidance document provides an important foundation and set of recommended working practices, and it can serve as the basis for patients, patient organizations, and regulatory authorities to continue developing and ultimately implementing meaningful collaborative interaction. Further refinement and elaboration of the guidance document, together with regulatory authorities, is suggested to arrive at a recognized code of practice for efficient interaction between regulatory authorities and patients/patient organizations.

## Author contributions

WS and DH drafted the article and the guidance document with significant input from AH, IK, CL, MM, and KW. All authors were contributors to the IMI project: European Patients' Academy on Therapeutic Innovation (EUPATI).

### Conflict of interest statement

The authors declare that the research was conducted in the absence of any commercial or financial relationships that could be construed as a potential conflict of interest.

## Footnotes

^1^To find out more visit www.eupati.eu/

^2^^*^Consumers are recognized as stakeholders in the healthcare dialogue. The scope of EUPATI focuses on patients rather than consumers this is reflected in the educational material and guidance documents.

^3^https://www.eupati.eu/regulatory-affairs/how-are-medicines-regulated/

^4^https://www.eupati.eu/webinar/webinar-guidance-patient-involvement-regulatory-processes/

^5^https://www.eupati.eu/regulatory-affairs/ema-regulatory-committees/

^6^https://www.eupati.eu/regulatory-affairs/medicines-regulation-in-europe/
